# Attractor Reconstruction for Quantifying the Arterial Pulse Wave Morphology During Device-Guided Slow Breathing

**DOI:** 10.1007/s13239-022-00628-0

**Published:** 2022-05-17

**Authors:** Carina Hörandtner, Martin Bachler, Walter Sehnert, Ines Mikisek, Thomas Mengden, Siegfried Wassertheurer, Christopher C. Mayer

**Affiliations:** 1 Center for Health & Bioresources, Medical Signal Analysis, AIT Austrian Institute of Technology GmbH, Giefinggasse 4, 1210 Vienna, Austria; 2 Institute for Analysis and Scientific Computing, Vienna University of Technology, Wiedner Hauptstr. 8-10, 1040 Vienna, Austria; 3 Institute for Clinical Research Sehnert, Dortmund, Germany; 4 Ines Mikisek Coaching, Frankfurt, Germany; 5 Kerckhoff Clinic, Rehabilitation, ESH Excellence Centre, Bad Nauheim, Germany

**Keywords:** Attractor reconstruction, Feature extraction, Arterial pulse waveform morphology

## Abstract

Attractor reconstruction is a new way to quantify the morphology of a cardiovascular waveform by plotting all data points in the three-dimensional phase space, generating a trajectory of overlapping loops. The aim of this study is to show the feasibility of an automatic approach to quantify pulse wave attractors from a device-guided breathing study, and to link attractor features to pulse waveform features. The recently developed feature extraction technique is applied to arterial pulse waveform data of 30 patients with treated hypertension. The patients performed a device-guided slow breathing exercise. The generated attractors were categorized into three different shapes: triangular attractors, bent attractors and attractors with overlapping arms. The average height of the attractors continuously and progressively dropped from 41.8 [35.4, 55.1] AU to 34.5 [25.4, 47.3] AU (*p* < 0.001) during the breathing exercise. We have shown that the novel approach to quantify pulse wave attractors is feasible and can be used to detect changes in the morphology of arterial pulse waveforms. Device-guided slow breathing exercise has a shrinking effect on the average height of the attractors, which may indicate a reduction in blood pressure.

**Supplementary Information:**

The online version contains supplementary material available at 10.1007/s13239-022-00628-0.

## Introduction

Cardiovascular disease (CVD) is one of the most common causes of death in Europe and in the United States.^20,22^ Early risk assessment is therefore of crucial importance and has been the subject of investigations for many decades. The examination of cardiovascular waveforms is a promising approach and has the potential for “prime time”.^7^

Up until now, various approaches to quantify pulse waveform and electrocardiography (ECG) data have been developed, whereas many methods only focus on averaged numerical values, such as heart rate or systolic and diastolic blood pressure (central or peripheral). This follows from the fact that usually a large quantity of data is collected, and the approach provides an easy way of summarizing some key features. Considering only a few data points, however, might miss changes of the waveform's morphology over time, which might provide valuable insight into the cardiovascular system. Other approaches using analyses in the time- or frequency-domain or by means of nonlinear approaches^14,23^ are as well promising but are not (yet) applicable in daily clinical routine.^1^ Therefore, new ways to provide useful information on the waveform's shape are needed.

One novel approach is attractor reconstruction which uses all data points of a recorded waveform and provides the possibility to assess the full waveform's morphology.^1,12^ This method applies a geometrical transformation to the original data, which is analysed to retrieve topological information about the dynamics of the observed data.^12^ It uses time delay coordinates based on Takens’ embedding theorem^19^ to plot signals, represented over a time axis, in a bounded reconstructed phase space. The method helps to gain deeper understanding of changes within a biological system and has been applied to other types of signals in order to investigate blood pressure data,^13^ respiration^18^ or EEG time series.^24^

Previous studies have already linked some attractor features to cardiovascular waveform features.^1,12^ The size of the reconstructed attractors, for example, can be connected to the pulse pressure, where a larger attractor corresponds to a signal with a high amplitude, i.e., a high pulse pressure. Depending on the signal, the attractors can vary in shape and orientation. Thus, in order to draw a conclusion about the waveform's morphology, proper and automatic quantification and classification of attractor features are important.

High blood pressure proved to be one of the most important risk factors for CVD.^6^ Besides medications and diets, previous studies have shown that slow breathing techniques, like meditation or yoga, reduce stress and have a positive impact on blood pressure.^3,9,10^ One mechanism that may lead to a reduction in blood pressure is vasodilation. This would also change the shape of the pulse waves, specifically in terms of relative ejection time and late systolic pulse waveform, which, in turn, would be an indication of decreasing afterload.^21^ Hence, a topic of interest is the influence of guided breathing exercises on attractor features and their changes over time.

Thus, the aim of this study is to demonstrate the feasibility of an automatic approach to quantify pulse wave attractors. Primarily, the different shapes of the attractors reconstructed from pulse waveform data acquired through photoplethysmography (PPG) and their connection to waveform features are analysed. As secondary outcome parameters, various features of the attractors and their change during 10 min of device-guided breathing and 5 min of unguided breathing are analysed. The main hypothesis is that the breathing exercise has a shrinking effect on the attractors and, thus, lower the overall pulse pressure.

## Materials and Methods

### Attractor Reconstruction

The attractor reconstruction method developed and described by Aston *et al*.^1^ and Nandi *et al*.^12^ is a new way to extract information from cardiovascular waveforms.

**Figure 1:**
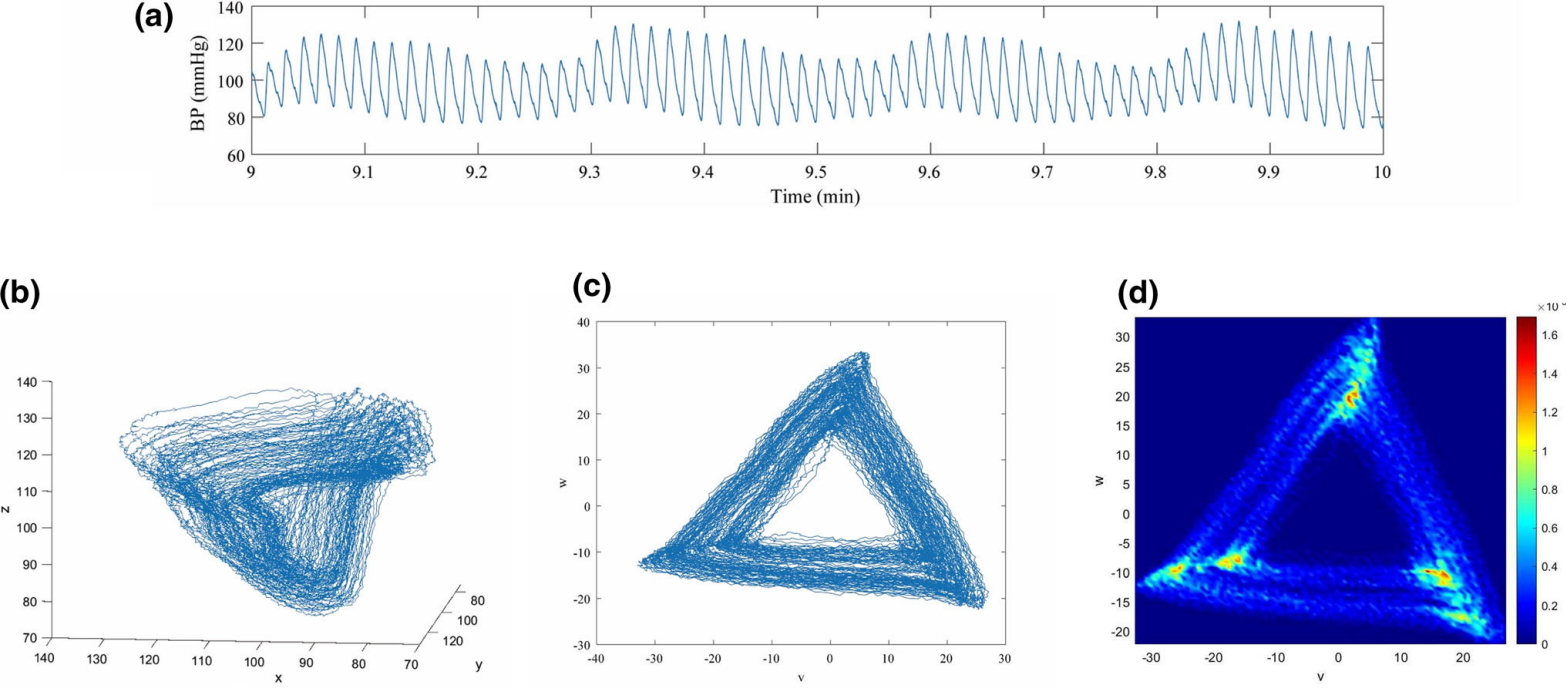
Attractor reconstruction using delay coordinates. (**a**) 60 s sample of pulse waveform data; (**b**) reconstructed attractor in the three dimensional phase space; (**c**) projection of the attractor onto a plane orthogonal to the vector (1, 1, 1); and (**d**) density plot of the two dimensional attractor.

Reconstructing a three-dimensional attractor using time delay coordinates based on Takens’ embedding theorem^19^ from a single signal (Fig. 1a) enables a compact representation of all data points in a time window of the entire sampled waveform. The time delay *τ >* 0 is chosen to be one third of the average heartbeat duration in the time window.^1^ Let *x*(*t*) define the given signal. Using the time delay *τ*, the coordinates of the three-dimensional attractor are calculated by

1 $$y(t) = x(t - \tau ),\quad z(t) = x(t - 2\tau ).$$

Plotting (*x*(*t*), *y*(*t*), *z*(*t*)) for all *t* in the time window generates the three-dimensional attractor (Fig. 1b). To eliminate the effect of a constant vertical shift in the signal *x*(*t*) and, consequently, in *y*(*t*) and *z*(*t*), the attractor is projected onto a plane orthogonal to the vector (1, 1, 1), as seen in Fig. 1c. This approach helps e.g., to remove respiratory induced baseline wander. The new set of coordinates (*u*, *v*, *w*) is defined as

2 $$u= \frac{1}{3}\left(x+y+z\right), v=\frac{1}{\sqrt{6}}\left(x+y-2z\right), w=\frac{1}{\sqrt{2}}\left(x-y\right).$$

This projection results in many overlapping lines with little detail visible. In order to enable the extraction of attractor features and their later comparison to features of the pulse waveform, a density is derived (Fig. 1d). This density matrix represents regions where the trajectory often returns to with a higher density and vice versa. Choosing the time delay *τ* to be on third of the average heartbeat duration results in a two-dimensional attractor with approximate 3-fold rotational symmetry, since the pulse waveform signal is approximately periodic.^1^

### Quantification

In order to detect changes in shape and variability of the pulse waveform over time, the attractor has to be quantified (see Online Supplement Fig. S1 for an overview of applied algorithm). In a first step, the quality of the data is examined to remove non-physiological artefacts. For this purpose, the maximum-to-minimum differences in sections of 4 s are calculated and segments containing outliers are removed from the signal. Then, the attractor is generated according to the previous section. Pre-processing is finally accomplished by application of a two-dimensional median filter to the density plot of the attractor to eliminate remaining noise.^15^

In the following, key points of the feature extraction method are provided (for details see Reference 8). It is based on image processing (i.e., Hough transform^4^ as a feature extraction technique to detect lines and their position and angle in the image) combined with the usage of the density matrix. First, the angle of rotation *θ* of the generated attractor is automatically calculated^8^ as the mean value of the feasible angles from the detected lines, so that its lower band becomes horizontal. The rotation provides information on the downstroke of the pulse wave^1,12^ and simplifies further calculations.

**Figure 2:**
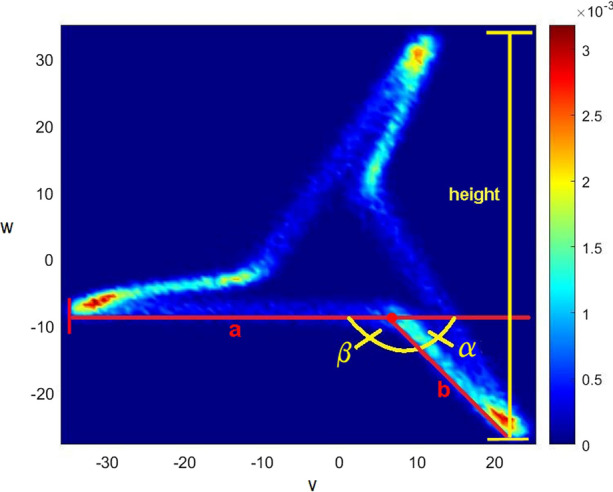
Attractor measurements.

One feature that catches one’s eye is that some attractor arms tend to split into two parts (Fig. 2), indicated by the angle *β* between the fragments. The angle *α* can be determined by rotating the attractor until the right fragment of the lower attractor arm becomes horizontal,^10^ and *β*_1_ = 180° − *α* can be determined. Due to the choice of the time delay *τ*, the attractor has 3-fold rotational symmetry^1^ and the process can be repeated on the rotated attractor (i.e., by 120° and 240°) to determine *β*_2_ and *β*_3_.

For the automatic measurement of the lengths of the attractor arm fragments (*a* and *b*, Fig. 2), again, Hough transform combined with the utilization of the density matrix and basic trigonometry is used.^8^ The length *b* of an attractor is set to zero, if two of the three attractor arm angles are above an empirically chosen threshold near 180°. For better comparison, the ratio *b*/*a* is calculated.

As the pulse pressure increases, the overall size of the attractor increases as well.^1,12^ Thus, the height *h* of the attractor as difference between the highest and lowest point in the density matrix is determined. Due to 3-fold rotation symmetry, the process can be repeated after rotating the attractor by 120° and 240°. By calculating the average of the three determined values, accuracy can be improved.

Furthermore, two time-domain parameters of the pulse wave shape were analysed, the normalized systolic peak time and the normalized dicrotic notch time. For details, see “Calculation of time-domain features” and Fig. S2 in the Online Supplement.

### Slow Breathing Study

In this paper, pulse wave data of a pilot study^2^ focusing on the effect of device-guided slow breathing quantified by change in pulse arrival time (PAT) is used. A ready-to-use wireless “Biosignal Explorer” system (biosignalsplux, Lisbon, Portugal) recorded ECG signals and pulse wave data through photoplethysmography (PPG) at the fingertip at a sampling rate of 256 Hz. No blood pressure measurements were taken because occlusion cuffs could stress the patients and thus distort the results of the relaxation exercise.^2,11^

Thirty patients with treated hypertension participated in the pilot study.^2^ Table 1 summarizes the baseline characteristics of the subjects. The protocol of the study was approved by the ethics committee of the Medical Association of Westphalia-Lippe and the University of Münster. The study was conducted in accordance with the Declaration of Helsinki. In order to facilitate free abdominal breathing, the examination was carried out in a comfortable sitting position. The patients were guided to the most uniform breathing with the aim to reach an average of 10 to 20 breaths per minute in everyday life or a maximum of 6 to 8 breaths per minute during respiratory therapy.

**Table 1:** Baseline characteristics of the subjects.

*N* (#)	30
Age (years)	62.9 (7.7)
Gender (#/#)	11 Females/19 males
Body height (cm)	174.4 (10.4)
Body weight (kg)	87.6 (18.9)
BMI	28.6 (4.7)
Arterial hypertension since (years)	9.5 [4, 15]
Systolic blood pressure (mmHg)	129.5 [124, 141]
Diastolic blood pressure (mmHg)	82 [76, 93]
Spontaneous respiratory rate (bpm)	13.6 (1.9)

Data expressed as mean (standard deviation), as median and interquartile range [IQR], or in absolute numbers^2^

The breathing guidance was implemented as a custom-made application for Android smartphones and tablets. During 10 min of exercise, a balloon shown on the screen symbolised the individual guided durations of expiration and inspiration of the users (see Fig. 1 in Reference 2). The guided breathing phase was followed by a 5 min unguided cooling down phase, where the balloon was hidden and the patients should try to continue to breathe calmly.

### Simulation of Idealised Pulse Wave Signals

In order to investigate how particular attractor features are associated with pulse waveform features, artificially generated signals with linear upstrokes and quadratic concave downstrokes and period 1 were considered.^1^ The function *x*(*t*) models these signals and is given by

3 $$x\left(t\right)=\left\{\begin{array}{ll}kt+d, & 0\le t\le T,\\ e{t}^{2}+ft+g, &T\le t\le 1.\end{array}\right.$$

Here, *k* is variable and denotes the gradient of the linear upstroke and *d* fixates the diastolic pressure value. The variables *e*, *f* and *g* influence the curvature of the quadratic downstroke and have to be chosen so that the signal stays continuous. The crest time, defined as the time frame between the pulse wave’s Nadir and its peak, is variable and is denoted as *T*, where *T* ϵ (0, 1).

In order to model a blood pressure level of about 120/80 mmHg and a crest time which matches *τ* =$\frac{1}{3}$, *k* was chosen to be 0.5, *d* was set to 80, *T* was set to $\frac{1}{3}$ and the variables modelling the downstroke *e*, *f* and *g* were chosen to be 0.0015, 0.7167 and 168, respectively. In order to get signals with a steeper upstroke, i.e., a blood pressure level of about 140/80 mmHg and *T <* $\frac{1}{3}$, the variables *k* was modified (*k* = 1*.*5567).

### Statistical Methods

A sliding window of 100 s was applied in 1-s steps to each of the 30 data sets to evaluate the change of the attractor features during the recording. The mean across all subjects along with the 95% confidence interval was calculated. The results were plotted as functions over time as difference from the baseline value (average of the first 3 min, i.e., 80 attractors) and as boxplots for direct comparison of baseline, end of guided breathing (average of minutes 7–10) and end of cooling phase (average of the last 3 min).

The extracted data was checked for normality using a Kolmogorov–Smirnov–Lilliefors-Test and was presented either with mean (standard deviation) or median and interquartile range, as appropriate. Because some data sets proved to be not normally distributed, nonparametric Friedman test was chosen to compare the three time frames and median and interquartile range (IQR) are reported. If the Friedman test revealed a (borderline) significant difference between baseline, end of guided breathing and end of cooling down, a multiple comparison test for pairwise *post hoc* comparison using Bonferroni correction was applied. Additionally, to compare categorical data for multiple measurements, a Cochran’s *Q* test was performed.^17^ Statistical significance was assumed at a 5% level. Computation was performed using MATLAB 2019b (The MathWorks, Inc., Natick, MA).

## Results

### Attractor Shapes

**Figure 3:**
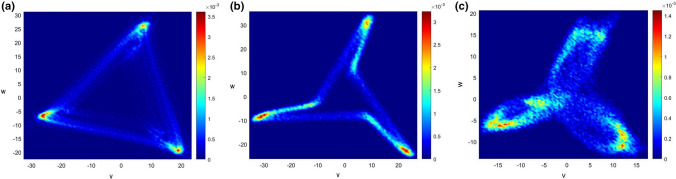
Different shapes of the attractors. (**a**) Triangular attractor; (**b**) bent attractor; and (**c**) attractor with overlapping arms.

Visual examination of 90 attractors, i.e., three for each subject (one at the baseline, one at the end of guided breathing and one at the end of the cooling down phase) revealed that the attractors can be categorized into three different shapes. Triangular attractors (Fig. 3a) are characterized by angles between arm fragments of almost 180°. As a consequence, *b* is set to zero, whereas bent attractors (Fig. 3b) have (at least two) angles distinctly below 180° and thus two attractor arm fragments (*a* and *b*). The third shape is distinguished by overlapping attractor arms (Fig. 3c) and cannot be detected by the applied method described in section *Quantification.*

### Breathing Exercise

**Figure 4:**
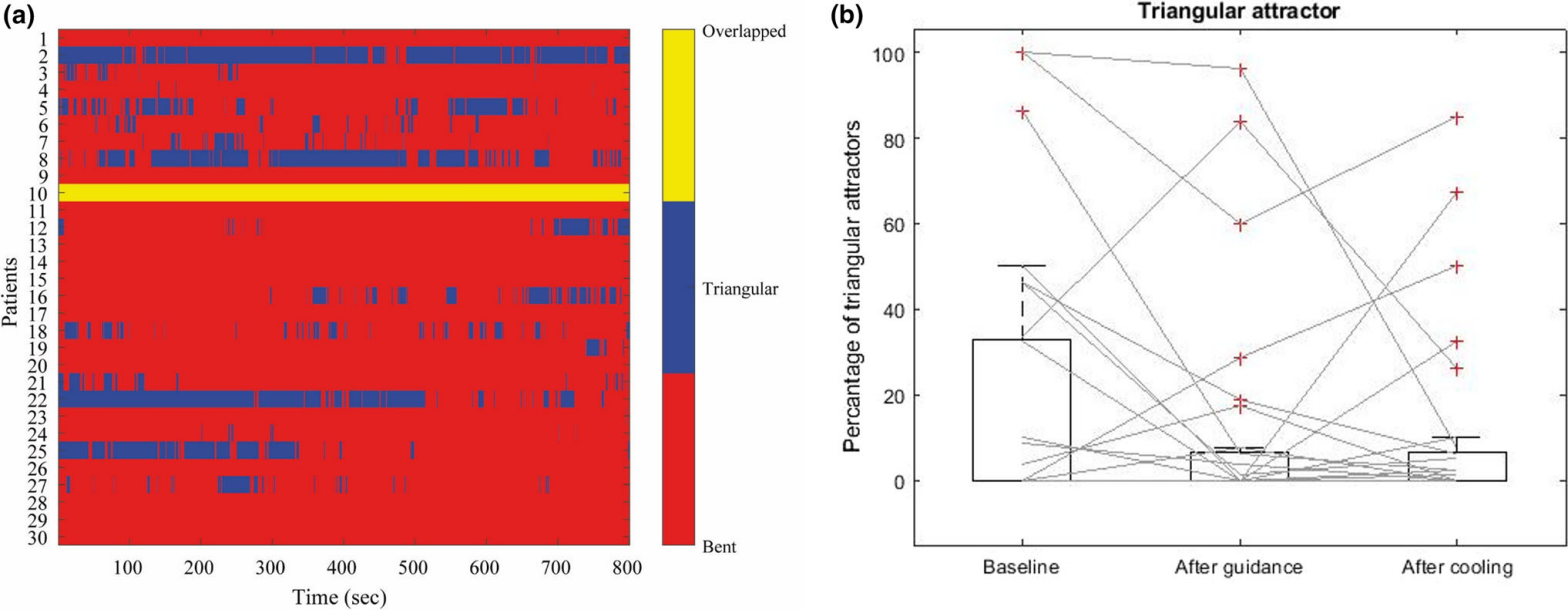
(**a**) Matrix representation of the evolution of the attractor shapes. (**b**) Boxplot of the percentage of triangular attractors at baseline, end of guided breathing, and end of cooling phase.

The frequency of the three different attractor shapes occurring during the recording is illustrated as a matrix in Fig. 4a. As mentioned, attractors with overlapping arms cannot be detected by the applied method. However, visual examination showed that this type of shape exists in especially one patient throughout the guided and unguided breathing. Of the remaining 29 patients participating in the study, 16 have a rather stable bent attractor frame, whereas triangle attractors predominate in 3 patients. To detect possible changes in shape during the recording, the percentage of triangle attractors at the three timeframes were compared. The application of the Friedman test yielded no significant changes (*p* = 0*.*34). We observed a median of 0% triangular attractors at each time frame, whereas the interquartile range dropped from 32.81 to 6.56% during the guided breathing exercise (Fig. 4b) indicating that more patients have a higher rate of triangular attractors during the first 3 min of recording. Cochran’s *Q* test comparing the appearance of bent and triangular attractors (13.79% of type triangle at baseline and 10.34% each at the end of breathing and at the end of cooling phase) confirmed that there are no significant chances (*p* = 0.85).

**Figure 5:**
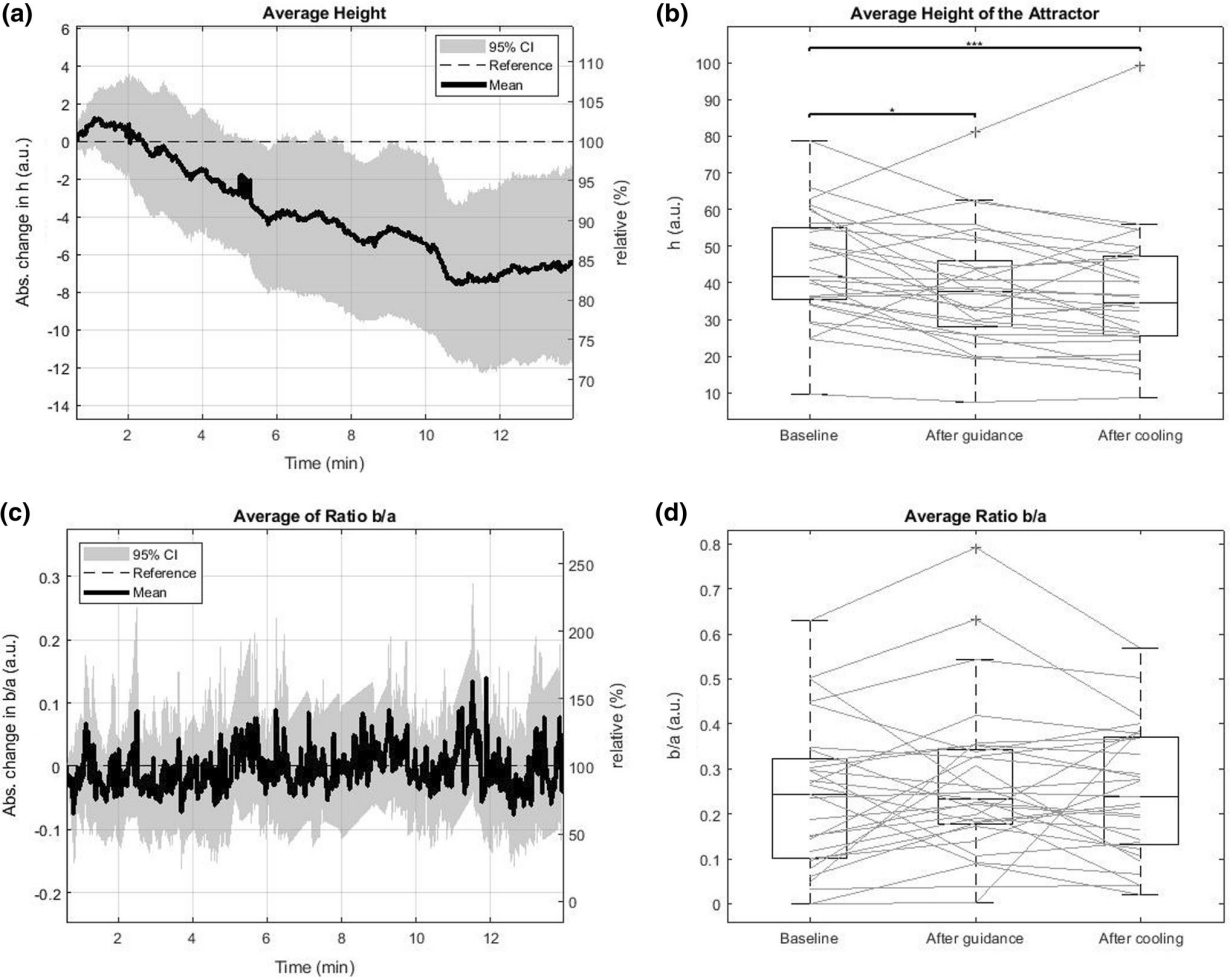
(**a**), (**c**) Evolution of the average height *h *and the average ratio *b*/*a* of the attractors during the recording. (**b**), (**d**) Boxplots of the average height *h *and the average ratio *b*/*a* of the attractors at baseline, end of guided breathing, and end of cooling phase. The stars indicate the level of significance, where one star means *p < *0.05 and three stars mean *p < *0.001.

After a small increase in the first minute, the average height of the attractor dropped during the guided exercise from 41.8 [35.4, 55.1] AU to 37.7 [28, 46] AU. In the cooling down phase, it decreased further to 34.5 [25.4, 47.3] AU, as shown in Figs. 5a and 5b. Friedman test comparing the three timeframes manifested overall significant changes (*p <* 0*.*001). *Post hoc* pairwise comparison revealed a significant drop during the guided breathing phase (*p* = 0.026), as well as between the baseline and the end of cooling phase (*p <* 0.001).

The ratio *b*/*a* displays a high fluctuation at each time step (Figs. 5c and 5d), where the median in interquartile range changed from 0.24 [0.10, 0.32] AU to 0.233 [0.178, 0.342] AU during the guided breathing phase before slightly increasing to 0.24 [0.13, 0.37] AU in the last 5 min of recording. Friedman test revealed a borderline significance (*p* = 0*.*08) and *post hoc* testing confirmed a borderline significant decrease during guided breathing (*p* = 0*.*077).

Results of the time-domain parameters can be found in the Online Supplement in section “Results of time-domain features” and in Fig. S3.

## Discussion

The aim of this study was to prove feasibility of a novel algorithm to quantify attractors generated from PPG data. Furthermore, the purpose of this paper was to examine how 10 min of device-guided breathing followed by 5 min of unguided breathing affect the size and shape of the attractors.

We found that the human pulse wave data, acquired through PPG at the fingertip during a slow-breathing study generated attractors with different shapes and orientations. Previous studies^1,12^ showed that a concave downstroke led to a clockwise rotation of the attractors. When applied to our data with more complex behaviour, i.e., more curvature changes, this trend was also seen. However, we noticed that further, unknown factors influence the complex interaction of pulse waveforms and attractors.

**Figure 6:**
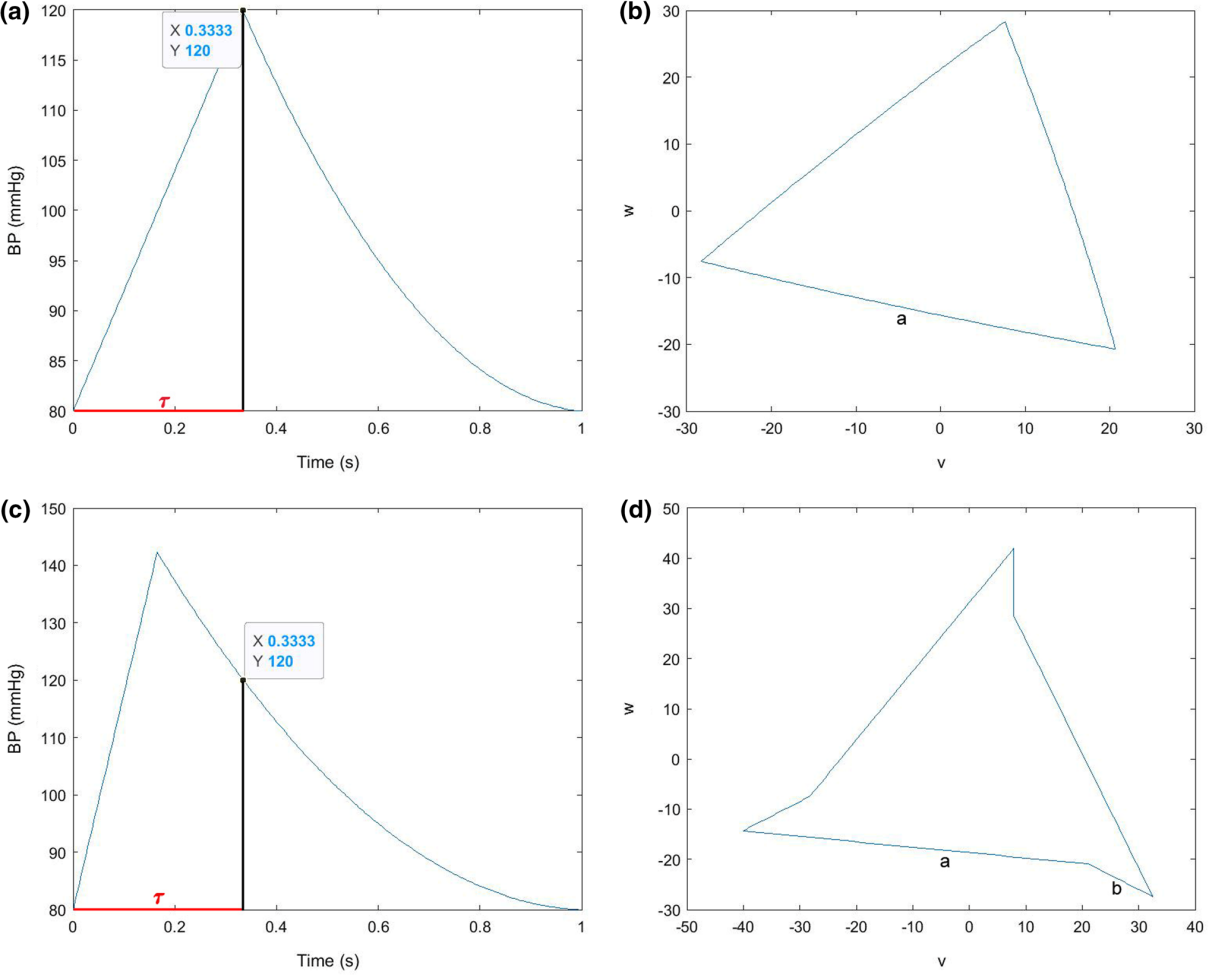
Artificially generated signals and corresponding attractors to illustrate the influence of *crest time*/*τ*. (**a**) Signal with *crest time*/*τ* = 1; (**b**) triangular attractor in the (*v*,* w*) plane; (**c**) signal with *crest time*/*τ* < 1; and (**d**) bent attractor in the (*v*,* w*) plane.

In this study, we categorized the attractors into three different shapes: triangular attractors, attractors with bends and attractors with overlapping arms, whereas the last form could not automatically be detected with the developed method and was only discovered after visual examination of the attractors. Investigations of artificially generated signals with linear upstrokes and parabolic quadratic downstrokes, i.e., no dicrotic wave visible, showed that if the ratio of the crest time to the time delay *τ* was 1 or close to 1 (Fig. 6a), the generated attractors are most likely to be categorized as triangular (Fig. 6b). This finding matches the results in Reference 1, where the impact of linear upstrokes and quadratic concave downstrokes with an approximate length of two thirds of the average heartbeat duration was investigated. Reduction of the crest time, i.e., *crest time*/*τ* < 1 (Fig. 6c), led to a division of the attractor sides into two fragments *a* and *b* (Fig. 6d), where *b*/*a* tended to be higher the smaller *crest time*/*τ*. Thus, it can be assumed that the ratio of crest time to *τ* is one of the factors influencing the shape of the attractor.

**Figure 7:**
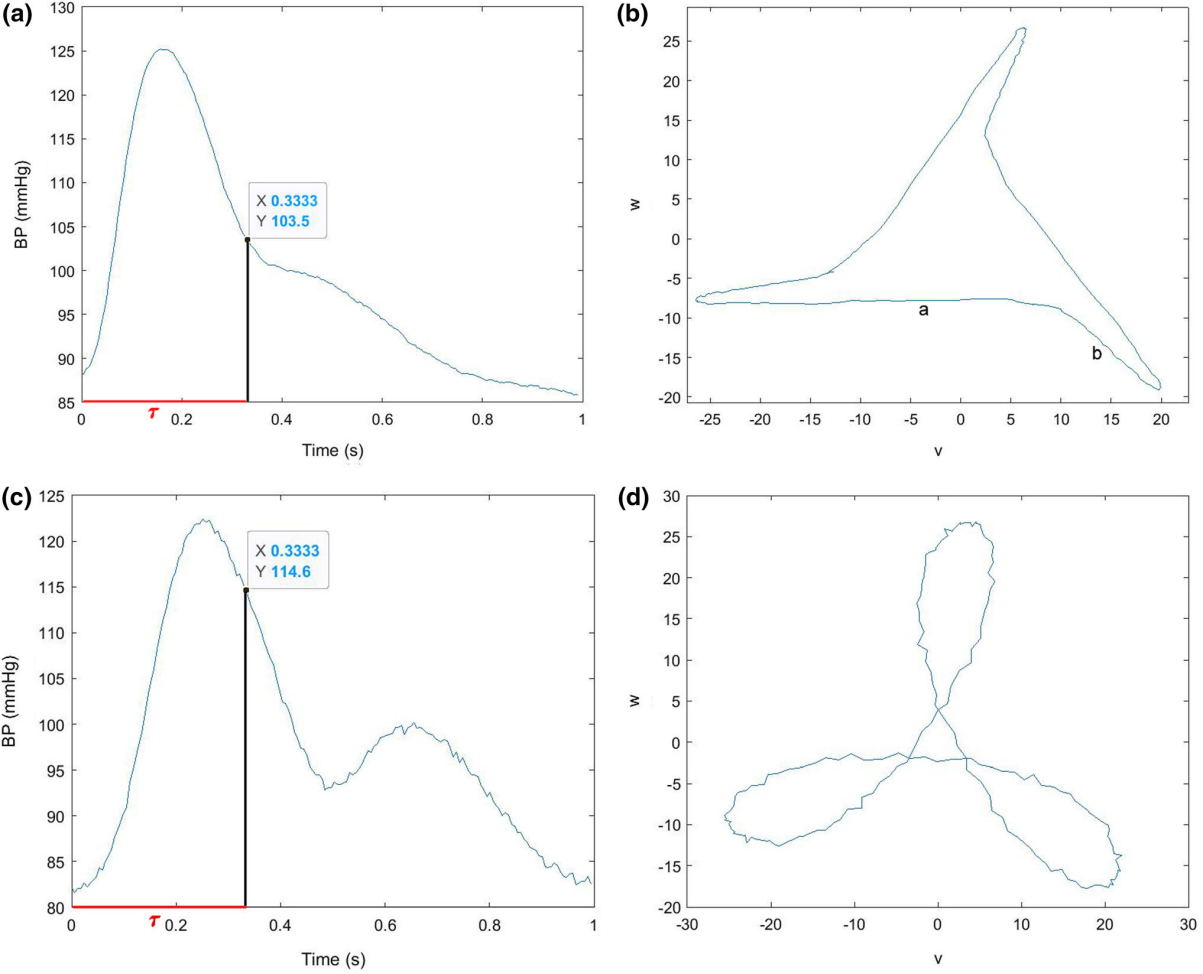
Pulse wave and corresponding attractor to illustrate the influence of curvature changes. (**a**) Signal with a small dicrotic wave and *crest time*/*τ* < 1; (**b**) bent attractor with a slightly negative angle of rotation *θ*; (**c**) signal with a distinct dicrotic wave and *crest time*/*τ* close to 1; and (**d**) attractor with overlapping sides.

Visual examination of physiological signals with a more complex structure, i.e., more curvature changes caused by the presence of a dicrotic notch and the following dicrotic wave, provided further insight into the intricate interaction of pulse waveforms and attractors. It was noticed that pulse waveforms with a visible dicrotic wave and crest *time*/*τ* < 1 (Fig. 7a) tended to be hardly rotated or even rotated in an anticlockwise direction (Fig. 7b). Attractors with overlapping sides seemed to be generated by pulse waves with a distinct dicrotic wave and *crest time*/*τ* close to 1 (Figs. 7c and 7d). In this study, this case appeared in only 1 of the 30 recordings investigated, indicating that this combination of features is not so common. Furthermore, this suggests that the position of the dicrotic notch and the dicrotic wave amplitude decisively influence the shape and orientation of the attractors. In order to ascertain specific correlations, further research is needed.

Even though more patients seemed to have a higher rate of triangular attractors during the first 3 min of recording than during the two other time frames, no significant trend throughout the breathing exercise can be seen. This means that the breathing technique had no significant impact on the shape of the attractor, or, in other words, the absence or presence of a dicrotic wave and the ratio of *crest time*/*τ* did not significantly change during the exercise. This is also reflected by the normalized systolic peak time in Fig. S3, which shows no change during the exercise. Some subjects enrolled in the study showed a stable behaviour of either triangular or bent attractors. For example, three patients predominantly showed triangle attractors. These three patients have PPG signals with hardly any dicrotic waves visible and a ratio of crest time to *τ* close to 1. The morphology of other patients’ signals seems to differ from these three signals. Other patients seemed to switch between the two shapes. One reason of the frequent shifts might be the fluctuation of the angles between the attractor arm fragments around the set threshold near 180°. Additionally, it is possible that image processing, i.e., Hough transform as a feature extraction technique, and the detection of the angle between the attractor arm fragments are subject to noise and hence, uncertainty. The quality of the given data sets is crucial for proper quantification of the resulting attractors, and it was discovered that remaining outliers and metrological effects, such as varying pressure on the recording device, had a negative impact on the accuracy.^8^ Furthermore, since attractors with overlapping arms cannot be detected automatically by the applied method, they might distort the results as well. The average ratio *b*/*a* fluctuated around the baseline value during the exercise but showed no definite trend.

**Figure 8:**
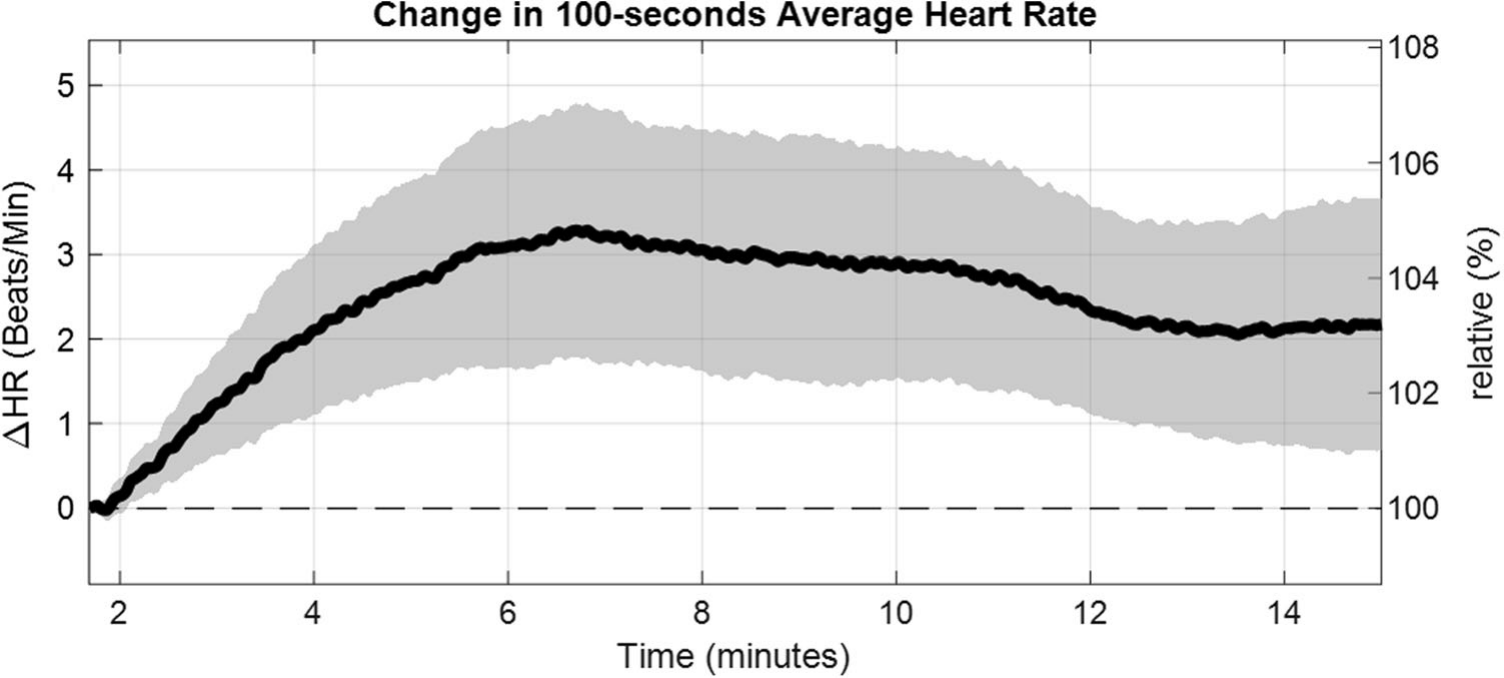
Difference of heart rate (HR) from baseline for all patients as function over time, smoothed using a 100 s moving average, shown as mean value with 95% confidence interval.

Due to its choice, the time delay *τ* it is directly correlated to the average heart rate. Bachler *et al*.^2^ investigated the difference of the heart rate from baseline for all patients as function over time (Fig. 8). It was discovered that guided breathing had a significant increasing effect on it, whereas unguided breathing decreased the average heart rate again. Although *τ* plays an important role in the construction of the attractor, it hardly influences the shape, size and orientation of the attractor.^1,12^

We found that the device-guided breathing exercise had a significant shrinking effect on the average height of the attractors which can be linked to a decrease in pulse pressure.^1,12^ Since previous studies revealed that relaxation techniques could lower blood pressure,^5,10,16^ it seems reasonable to connect the decline in pulse pressure to a decrease in blood pressure. These findings are in line with the results from Bachler *et al*.,^2^ where the PAT showed an inverse but similar behaviour and significantly increased during the breathing exercise which can be linked to a decrease in mean blood pressure. The PAT is measured *via* two signals (a 1-lead ECG and finger plethysmography). Hence, attractor reconstruction could potentially simplify the recording mode by using only PPG data with similar outcome.

The first issue worth mentioning is that we set the sliding window length to 100 s and did not vary it. However, comparing the results of different time windows might provide more information on the influence of the selected length. Furthermore, we only had one development and testing data set. In order to verify our results and rule out artefacts, an independent data set would have been of advantage. Another limitation of the pilot study^2^ is that no blood pressure data was recorded during the exercise. Thus, a definite statement regarding the effect of device-guided breathing on blood pressure is not possible. Furthermore, all patients enrolled in this study suffer from essential hypertension. In order to compare and evaluate the effect of guided breathing, a control group of healthy individuals would be of advantage. Another limitation is that the pulse waveform data of the subjects were recorded only for 5 min past the end of the guided breathing. However, a longer recording period after guidance might reveal further effects of slow breathing on the cardiovascular system.

In this study, we have shown that attractors generated from PPG data can be categorized into three different shapes, whereas the developed method only properly detected triangular and bent attractors and could not appropriately quantify attractors with overlapping arms. We discovered that the crest time and the position of the dicrotic notch and the dicrotic wave amplitude essentially influence the shape and orientation of the attractors. Furthermore, application to a device-guided breathing study showed that the attractor reconstruction method is feasible for detection of pulse pressure changes and might provide insight into blood pressures changes as well. As assumed, the slow breathing exercise had a shrinking effect on the average height of the attractors which can be linked to a decrease in pulse pressure.

## Supplementary Information

Below is the link to the electronic supplementary material.

Supplementary file1 (PDF 255 kb)

## References

[1] Aston PJ, Christie MI, Huang YH, Nandi M (2018). Beyond HRV: attractor reconstruction using the entire cardiovascular waveform data for novel feature extraction. Physiol. Meas..

[2] Bachler M, Sehnert W, Mikisek I, Wassertheurer S, Mengden T (2020). Non-invasive quantification of the effect of device-guided slow breathing with direct feedback to the patient to reduce blood pressure. Physiol. Meas..

[3] Brook R, Appel L, Rubenfire M, Ogedegbe G, Bisognano J, Elliott W, Fuchs F, Hughes J, Lackland D, Staffileno B, Townsend R, Rajagopalan S (2013). Beyond medications and diet: alternative approaches to lowering blood pressure a scientific statement from the American Heart Association. Hypertension.

[4] Duda R, Hart P (1972). Use of the Hough transformation to detect lines and curves in pictures. Commun. ACM.

[5] Elliott W, Izzo J (2006). Device-guided breathing to lower blood pressure: case report and clinical overview. Medscape Gen. Med..

[6] Fuchs FD, Whelton PK (2020). High blood pressure and cardiovascular disease. Hypertension.

[7] Hametner B, Wassertheurer S (2017). Pulse waveform analysis: is it ready for prime time?. Curr. Hypertens. Rep..

[8] Hörandtner, C., M. Bachler, S. Wassertheurer, F. Breitenecker, and C. C. Mayer. Pulse wave analysis by quantified reconstructed attractors. *SNE* 32(2):69–78, 2022.

[9] Irvine MJ, Johnston DW, Jenner DA, Marie GV (1986). Relaxation and stress management in the treatment of essential hypertension. J. Psychosom. Res..

[10] Joseph CN, Porta C, Casucci G, Casiraghi N, Maffeis M, Rossi M, Bernardi L (2005). Slow breathing improves arterial baroreflex sensitivity and decreases blood pressure in essential hypertension. Hypertension.

[11] Liu Q, Yan B, Yu CM, Zhang YT, Poon C (2013). Attenuation of systolic blood pressure and pulse transit time hysteresis during exercise and recovery in cardiovascular patients. IEEE Trans. Biomed. Eng..

[12] Nandi M, Venton J, Aston PJ (2018). A novel method to quantify arterial pulse waveform morphology: attractor reconstruction for physiologists and clinicians. Physiol. Meas..

[13] Narayana Dutt, D., and S. M. Krishnan. Application of phase space technique to the analysis of cardiovascular signals. In: Proceedings of IEEE EMBS Conference, Atlanta, USA, 1999.

[14] O’Rourke MF (2009). Time domain analysis of the arterial pulse in clinical medicine. Med. Biol. Eng. Comput..

[15] Paranjape RB, Bankman IN (2009). Chapter 1—fundamental enhancement techniques. Handbook of Medical Image Processing and Analysis.

[16] Rau H, Bührer M, Weitkunat R (2003). Biofeedback of r-wave-to-pulse interval normalizes blood pressure. Appl. Psychophysiol. Biofeedback.

[17] Sheskin DJ (2007). Handbook of Parametric and Nonparametric Statistical Procedures.

[18] Small M, Judd K, Lowe M, Stick S (1999). Is breathing in infants chaotic? Dimension estimates for respiratory patterns during quiet sleep. J. Appl. Physiol..

[19] Takens F, Rand D, Young LS (1981). Detecting strange attractors in turbulence. Dynamical Systems and Turbulence, Warwick 1980.

[20] Timmis A (2019). European Society of Cardiology: cardiovascular disease statistics 2019. Eur. Heart J..

[21] Tusman G, Acosta CM, Pulletz S, Böhm SH, Scandurra A, Arca JM, Madorno M, Sipmann FS (2019). Photoplethysmographic characterization of vascular tone mediated changes in arterial pressure: an observational study. J. Clin. Monit. Comput..

[22] Virani S (2021). Heart disease and stroke statistics—2021 update. Circulation.

[23] Voss A, Schulz S, Schroede R, Baumert M, Caminal P (2009). Methods derived from nonlinear dynamics for analysing heart rate variability. Philos. Trans. R. Soc. A.

[24] Wang X, Meng J, Tan G, Zou L (2010). Research on the relation of EEG signal chaos characteristics with high-level intelligence activity of human brain. Nonlinear Biomed. Phys..

