# Supplementary material for: Attractor Reconstruction for Quantifying the Arterial Pulse Wave Morphology During Device-Guided Slow Breathing
Source: Cardiovasc Eng Technol. 2022 May 17;13(6):939–49. doi: 10.1007/s13239-022-00628-0 (PMC9750906; doi:10.1007/s13239-022-00628-0)
Supplement: Supplementary file 1 — Supplementary file1 (PDF 255 kb) [file 13239_2022_628_MOESM1_ESM.pdf]

Supplement

Attractor reconstruction for quantifying the arterial pulse wave morphology during device-guided slow breathing

Carina Hörandtner[1,2], Martin Bachler[1], Walter Sehnert[3], Ines Mikisek[4], Thomas Mengden[5], Siegfried Wassertheurer[1] and Christopher C. Mayer[1,2]

[1] AIT Austrian Institute of Technology GmbH, Center for Health & Bioresources, Biomedical Systems, Giefinggasse 4, 1210 Vienna, Austria

[2] Vienna University of Technology, Institute for Analysis and Scientific Computing, Wiedner Hauptstr. 8-10, 1040 Vienna, Austria

[3] Institute for Clinical Research Sehnert, Dortmund, Germany

[4] ines mikisek coaching, Frankfurt am Main, Germany

[5] Kerckhoff Clinic, Rehabilitation, ESH Excellence Centre, Bad Nauheim, Germany

Flow chart representation of the developed algorithm

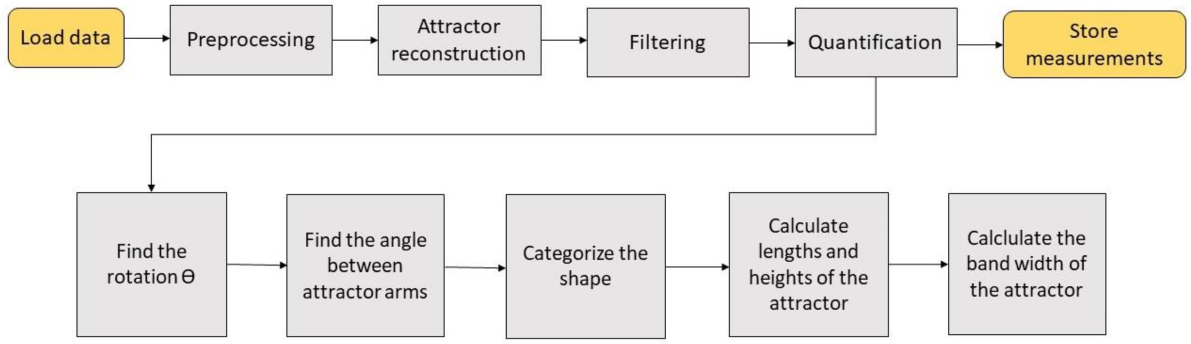

Figure S1: Flow chart representation of the applied algorithm.

Calculation of time-domain features

The following time-domain features are extracted from the recorded PPG signals on a beat-by-beat basis and averaged over 100 seconds:

Normalized systolic peak time (t_{sys} / t_T): This wave shape parameter of the pulse waves is extracted using pulse wave analysis, as shown in Figure S1 [1]. The systolic peak time is defined as the time from the onset point of the pulse wave to the maximum of the signal within the same heartbeat. This time is then normalized with respect to the duration of the whole pulse wave as t_{sys} / t_T.

Normalized dicrotic notch time (t_{notch} / t_T): Similarly, the dicrotic notch is defined as the local signal minimum shortly after t_{sys}, or as the maximum curvature point, depending on the shape of the wave. It is also normalized with respect to the duration of the whole pulse wave as t_{notch} / t_T [1].

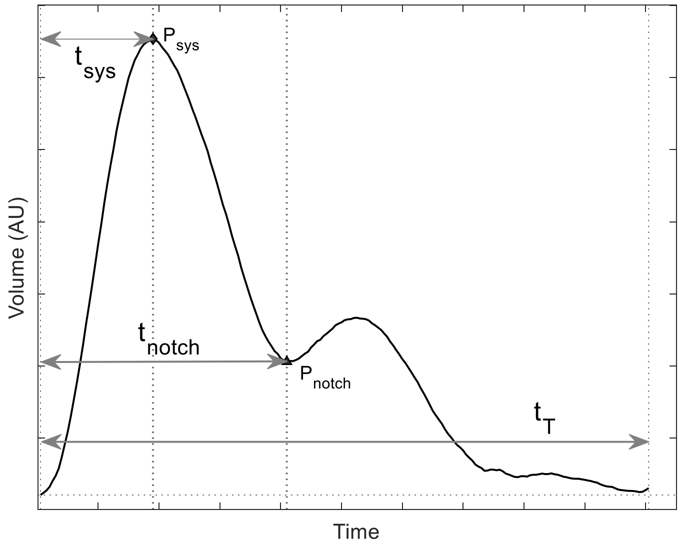

Figure S2: Parameters calculated from the wave shape using pulse wave analysis: one pulse wave with marked timings.

Results of time-domain features

The analysis of the normalized wave shape parameters systolic peak time t_{sys} / t_T and dicrotic notch time t_{notch} / t_T reveals some changes during the device guided breathing and cooling phase, as shown in Figure S3. As the heart rate increases (see Figure 8), the total pulse duration t_T naturally shows an inverse effect and decreases. The normalized systolic peak time t_{sys} / t_T shows no trend, indicating that the rise time of the pulse wave shape stays constant relative to the total pulse duration. The normalized dicrotic notch time t_{notch} / t_T shows a steady increase during the first 7 minutes of the device guided breathing exercise, followed by a very slow decrease for the remainder of the exercise, and almost returns to baseline during the cooling phase. This indicates a broadening of the wave shape in the diastolic phase, especially during the first 7 minutes of the exercise.

The normalized t_{notch} / t_T increased by 0.01 ($p<0.01$, SD 0.02) during the first 7 minutes of guided breathing, and in total by 0.007 ($p<0.05$, SD 0.02) by the end of the guided breathing exercise after 10 minutes, compared to baseline.

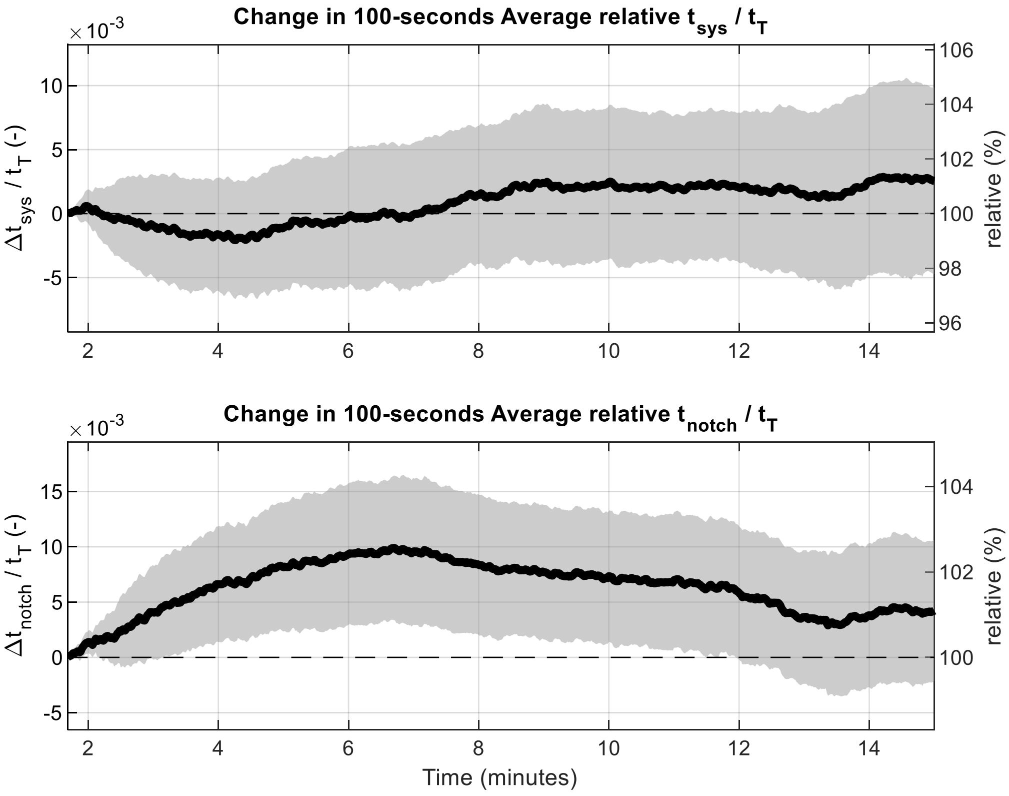

Figure S3: Changes of wave shape parameters during the exercise.

References

[1] Chorherr, P. (2019). Development and evaluation of an arterial pulse waveform analysis algorithm. Master Thesis, TU Wien.
